# Improvement in the physiological and biochemical performance of strawberries under drought stress through symbiosis with Antarctic fungal endophytes

**DOI:** 10.3389/fmicb.2022.939955

**Published:** 2022-08-25

**Authors:** Luis Morales-Quintana, Mario Moya, Rómulo Santelices-Moya, Antonio Cabrera-Ariza, Claudia Rabert, Stephan Pollmann, Patricio Ramos

**Affiliations:** ^1^Multidisciplinary Agroindustry Research Laboratory, Instituto de Ciencias Biomédicas, Facultad Ciencias de la Salud, Universidad Autónoma de Chile, Talca, Chile; ^2^Plant Microorganism Interaction Laboratory, Centro del Secano, Facultad de Ciencias Agrarias y Forestales, Universidad Católica del Maule, Talca, Chile; ^3^Centro del Secano, Facultad de Ciencias Agrarias y Forestales, Universidad Católica del Maule, Talca, Chile; ^4^Centro de Investigación de Estudios Avanzados del Maule, Vicerrectoría de Investigación y Postgrado, Universidad Católica del Maule, Talca, Chile; ^5^Instituto de Ciencias Biomédicas, Facultad Ciencias de la Salud, Universidad Autónoma de Chile, Temuco, Chile; ^6^Centro de Biotecnología y Genómica de Plantas, Instituto Nacional de Investigación y Tecnología Agraria y Alimentaria, Universidad Politécnica de Madrid, Pozuelo de Alarcón, Spain; ^7^Centro de Biotecnología de los Recursos Naturales, Facultad de Ciencias Agrarias y Forestales, Universidad Católica del Maule, Talca, Chile

**Keywords:** root fungal endophytes, drought, Antarctic microorganisms, functional symbiosis, strawberry

## Abstract

Strawberry is one of the most widely consumed fruit, but this crop is highly susceptible to drought, a condition strongly associated with climate change, causing economic losses due to the lower product quality. In this context, plant root-associated fungi emerge as a new and novel strategy to improve crop performance under water-deficiency stress. This study aimed to investigate the supplementation of two Antarctic vascular plant-associated fungal endophytes, *Penicillium brevicompactum* and *Penicillium chrysogenum*, in strawberry plants to develop an efficient, effective, and ecologically sustainable approach for the improvement of plant performance under drought stress. The symbiotic association of fungal endophytes with strawberry roots resulted in a greater shoot and root biomass production, higher fruit number, and an enhanced plant survival rate under water-limiting conditions. Inoculation with fungal endophytes provokes higher photosynthetic efficiency, lower lipid peroxidation, a modulation in antioxidant enzymatic activity, and increased proline content in strawberry plants under drought stress. In conclusion, promoting beneficial symbiosis between plants and endophytes can be an eco-friendly strategy to cope with drought and help to mitigate the impact of diverse negative effects of climate change on crop production.

## Introduction

Climate change is affecting food security, and global warming is challenging agricultural production all around the world. The occurrence of intense and longer periods of drought, together with high temperatures (Grossiord et al., 2020), reduce crop yield and fruit quality. This is particularly important in countries like Chile with an export-based economy. The scarcity of water resources is the main cause of crop loss worldwide (Munns and Tester, 2008; Ksoury et al., 2015), which will soon become even more severe as desertification progressively affects more and more regions of the world (Vinocur and Altman, 2005). All this affects agriculture worldwide, mainly because crop yields are reduced (Leisner, 2020).

Drought stress is a global problem; however, it is a particularly important case in developing countries, where these abiotic stresses severely limit crops production (Gleick, 2000; Zhou et al., 2007). Thus, drought stress is produced when the water availability in the soil is reduced below critical levels that impairs normal plants’ growth and development (Ramakrishna and Ravishankar, 2011). Plants display a suite of mechanisms to mitigate the negative impact of stress in general, including water deficit (Taìtrai et al., 2016). Different authors have described that the mechanisms can be boosted by symbiosis with microorganisms (Wani et al., 2015; Barrera et al., 2020; Hereme et al., 2020; Morales-Quintana et al., 2021). In this context, one of the most extreme environmental conditions for life is in Antarctica, including low water availability, high UV-B radiation, extremely low temperatures, and saline soils (Convey et al., 2014), and despite this, two vascular plants inhabit this hostile environment, namely, *Deschampsia antarctica* and *Colobanthus quitensis* (Parnikoza et al., 2007). Additionally, several studies have demonstrated that endophytic microorganisms, such as fungi and bacteria, isolated from Antarctic plants allow other hosting plants tolerate environmental stresses (Molina-Montenegro et al., 2016; Acuña-Rodríguez et al., 2019, 2022). Two species of fungi, *Penicillium chrysogenum* and *Penicillium brevicompactum*, were identified in Antarctic plants, and their functional role was investigated in their native host plant as well as in other plant species, such as tomato and lettuce (Molina-Montenegro et al., 2020). Recently, the effect of two Antarctic root endophytes, *Penicillium rubens* and *Penicillium bialowienzense*, was evaluated in the response of blueberry plants under cold and drought conditions (Acuña-Rodríguez et al., 2022).

On the other hand, strawberry (*Fragaria* × *ananassa* Duch.) plants have shallow roots, which make it a very sensitive crop to soil water deficit, specifically during flowering and fruit ripening. Strawberry is among the most widely consumed fruit in the world. The allo-octoploid cultivated strawberry is a financially important fruit. In 2012, the world strawberry harvested area was 241,000 ha with 4,516,810 tons of fruit production.^1^ The coast of Maule and the metropolitan are the two main strawberry growing regions in Chile with 80% of the Chilean production.^2^ In soils with low water availability, strawberry crops show a significant decrease in physiological parameters (Mozafari et al., 2019; Cai et al., 2020), which causes a significant reduction in yield, because the plant has a shallow root system, a large leaf surface, and a high water content in the fruit (Liu et al., 2007; Nezhadahmadi et al., 2015).

Previous studies reported the symbiosis of the *Fragaria* genus with various types of fungi (Rivera-Chaìvez et al., 2012; Salgado-Barreiro et al., 2012). Yokoya et al. (2017) isolated 61 different endophytic fungi from samples obtained from *Fragaria vesca* plants collected from different locations, demonstrating the plant’s ability to associate with a wide range of endophytic fungal groups. In this sense, Watanabe and Inoue (1980) reported that the most predominant genus of fungi in strawberry plant roots is *Penicillium* spp.

In this article, we report the ability of two Antarctic fungal endophytes, *P. chrysogenum* and *P. brevicompactum*, to improve the performance of strawberry plants exposed to drought stress. Biochemical and physiological parameters suggest that fungal endophytes can support the plant to deal with the water deficit. Moreover, this symbiosis avoids detrimental effects on the yield and quality of fruits.

## Materials and methods

### Plants and fungal material

Eighty-four plants of *Fragaria* × *ananassa* (Strawberry) cultivars ‘Aromas’ were obtained from a commercial company and grown in 1-L pots with a standard peat–perlite (1:1) mixture in greenhouse at the Universidad Católica del Maule, Maule Region, Chile. For the obtention of plants free of microbial endophytes (E−), half of them were treated with 2 g L^–1^ of the commercial fungicide Benlate (DuPont, Wilmington, DE, United States), based on previous reports (Ramos et al., 2018; Barrera et al., 2020; Hereme et al., 2020). In addition, all plants were treated with systemic broad-spectrum antibiotic rifampicin (50 μg mL^–1^) to remove any bacteria (Ramos et al., 2018; Barrera et al., 2020; Hereme et al., 2020). Water was used as the control for the fungicide treatment in E + plants. After 2 weeks, five plants, each of E + and E−, were randomly inspected to verify the presence and/or absence of endophytes based on Barrera et al. (2020).

**FIGURE 1:**
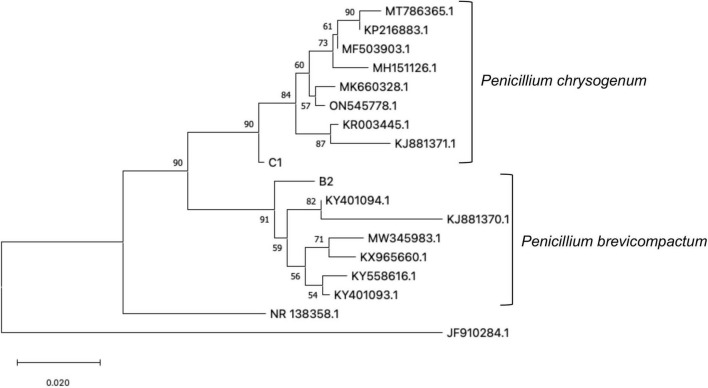
Phylogenetic relationship tree between 45 sequences of endophytic fungi based on the ITS rDNA sequences NCBI sequences: KR003445.1 *Penicillium chrysogenum*; MK660328.1 *Penicillium chrysogenum*; ON545778.1 *Penicillium chrysogenum*; MH151126.1 *Penicillium chrysogenum*; MF503903.1 *Penicillium chrysogenum*; MT786365.1 *Penicillium chrysogenum*; KP216883.1 *Penicillium chrysogenum*; MW345983.1 *Penicillium brevicompactum*; KY401094.1 *Penicillium brevicompactum*; KY558616.1 *Penicillium brevicompactum*; KY401093.1 *Penicillium brevicompactum*; KX965660.1 *Penicillium brevicompactum*; KJ881370.1 *Penicillium brevicompactum*; KJ881371.1 *Penicillium chrysogenum*; JF910284.1 *Penicillium minioluteum*; NR_138358.1 *Penicillium murcianum*. Values in the branch nodes correspond to bootstrap.

For the inoculation, two Antarctic fungal endophytes, previously identified by Molina-Montenegro et al. (2020), were isolated from *C. quitensis* and *D. antarctica* plants, each randomly collected from different locations near Henryk Arctowski Polish station in Admiralty Bay on King George Island, Antarctic Peninsula (Rabert et al., 2020). Sequencing of ITS from DNA isolated from axenic fungal strains was performed to verify the identity of *P. chrysogenum* (Genbank accession number: KJ881371) and *P. brevicompactum* (Genbank accession number: KJ881370)^3^ based on Molina-Montenegro et al., 2016. The fungal inoculum was prepared as a 1:1 mix of the two strains isolated and identified as C1 and B2 (Figure 1) according to Molina-Montenegro et al. (2020). The rhizosphere of each individual plant was inoculated with 3 mL of concentrated solution with the mix of spores (1 × 10^6^–1 × 10^7^ spores mL^–1^) from each fungal endophyte. The inoculation was repeated 15 days later to ensure an effective association of fungi with the plants. The symbiosis was verified as previously described (Barrera et al., 2020). Two plants of each treatment group were randomly selected to verify the presence or absence of endophytes in roots, staining a section of the root with trypan blue in an acid glycerol solution and observing it under a light microscope (iScope, Euromex, Netherland). Additionally, strawberry roots from the inoculated group were re-checked for the presence of the fungal endophyte at the end of the treatments (after 60 days) (See Supplementary Figure 1).

### Experimental design

One hundred strawberry plants with (E+) and without (E−) endophytic fungi were subjected to 60 days of contrasting water irrigation regimens. Plants were divided into four treatments: (1) 100% of the water irrigation [water-holding capacity (WHC)] (400 ml) (W + E−), (2) 50% of WHC (200 ml) (W−E−), (3) 100% of WHC plus root-endophytes inoculation (W + E +), and (4) 50% of WHC plus root-endophytes inoculation (W−E +). The amount of water to drought condition was established based on a gravimetric methodology (Erdogan et al., 2016). Briefly, the pots were watered until saturation and freely drained until there was no change in the weight. The difference between this weight and soil dry weight was used to calculate 100% of WHC. Then, 100 and 50% of WHC for plant cultivation were periodically determined gravimetrically and maintained by daily irrigation.

### Skin fruit color determination

Fruits without external damage were examined for their skin color. Six strawberries per treatment were chosen randomly for color measurement at the equator of fruit. The surface color of the fruits from different treatments was characterized using a colorimeter model CR-400 (Konica Minolta, Tokyo, Japan) and expressed in the Hunter scale (L*, a*, and b*). The CIELAB color representation was generated by a Nix Color converter.^4^

### Physiological and biochemical determinations

#### Photosynthetic performance, chlorophylls and carotenoids content, free proline concentration, and membrane damage

The photochemical efficiency of PSII (Fv/Fm) was estimated in accordance with Maxwell and Johnson (2000). For this, plants were assessed for each condition (time of exposure and presence/absence of endophyte) using a pulse-modulated amplitude fluorimeter (FMS 2, Hansatech, Instrument Ltd., Norfolk, United Kingdom).

Chlorophyll (Chl) and carotenoids (Car) were extracted from shoot tissue (100 mg) with 80% acetone and determined at the following wavelengths: 663 nm (Chl *a*), 646 nm (Chl *b*), and 470 nm (Car) and calculated by the spectrophotometric method described by Lichtenthaler and Wellburn (1983).

The free proline concentration evaluation was determined following the protocol of Bates et al. (1973). In brief, shoot tissue (100 mg) was frozen in liquid nitrogen and grounded in 1.2 ml of 3% sulfosalicylic acid. Then, the homogenate was centrifuged at 16,000 g at room temperature for 20 min. An aliquot of the supernatant (1 ml) was mixed with 2 ml ninhydrin reagent [2.5% ninhydrin in glacial acetic acid—distilled water—85% orthophosphoric acid (6:3:1)]. The reaction mixtures were kept in a water bath at 90 °C for 1 h to develop the color. Samples were put on ice, and 2 ml toluene was added to separate the chromophore. The toluene phase was separated and the absorbance was read at 525 nm (Multiskan SkyHigh Microplate Spectrophotometer, Thermo Fisher Scientific). The proline content was calculated using a standard proline curve.

Additionally, to assess whether the fungal endophytes protect plants from water stress, lipid peroxidation, photochemical efficiency, and synthesis of proline were evaluated in shoot tissue of strawberry plants exposed to the condition of simulated drought after 60 days. Lipid peroxidation leads to an increase in the levels of malondialdehyde (MDA) and is considered a useful index for oxidative cell damage (Møller et al., 2007). Shoot tissue (100 mg) was homogenized and the MDA content was evaluated following the procedure previously reported by Barrera et al. (2020).

#### Antioxidant enzymatic assays

Antioxidative enzyme activity was determined in shoot tissue. Briefly, 500 mg of leaves (fresh weight) was ground in liquid nitrogen and the powder was transferred to tubes containing pre-chilled 50 mmol L^–1^ phosphate buffer (pH 7.8, containing 1% PVP) and centrifuged at 4°C, 10,000 r min^–1^ for 20 min. Then, 5 ml of the supernatant was transferred to new tubes for superoxide dismutase (SOD), catalase (CAT), peroxidase (POD), and ascorbate peroxidase (APX) activity assays according to Erdogan et al. (2016). Determinations were performed in biological triplicates.

#### Statistical analysis

For the determination of MDA, Fv/Fm, proline content, chlorophylls, carotenoid, biomass, fruit weight, the skin color of the fruit, and diameter, two-way ANOVA was used with the factors water condition (W) and presence of endophytic fungi (E). Normality and homoscedasticity of the data were confirmed before the ANOVA test. Tukey’s HSD multiple comparisons analysis was performed to evaluate significant differences between treatments. All analyses were performed using GraphPad Prism 8 (GraphPad Software Inc., San Diego, CA, United States).

## Results

### Root colonization, biomass production, and survival of strawberry plants

Under drought stress, microscopic visualization revealed that Antarctic fungal endophytes successfully colonized the root of strawberry plants at 60 days post inoculation (Supplementary Figure 1). Spores and hyphae were able to grow inter- and intra-cellularly in both, well-watered and drought-stressed strawberry roots.

**FIGURE 2:**
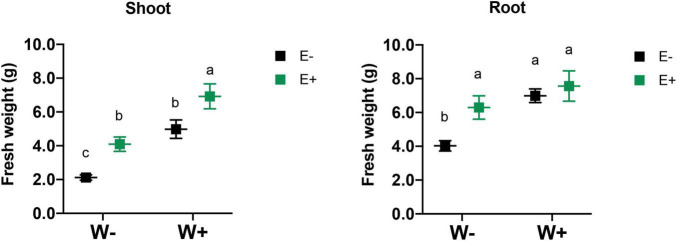
Effect of drought stress and fungal inoculation on shoot and root biomass production of inoculated and non-inoculated strawberry plants Different letters indicate significant differences (*P* < 0.05; two-way ANOVA). Bars represent means ± SE from three independent experiments.

Biomass production and survival of plants with and without fungal endophytes were evaluated under drought stress. The survival plant rate was calculated by counting the number of plants that survived divided by the number of plants originally established for each treatment and then multiplied by 100 to express the as the percentage of survival. As was expected, the water-deficient treatment strongly reduced the shoot biomass production in plants without endophytes (W−E−) compared to well-watered plants (W+E−) (See Supplementary Figure 2). When the inoculated plants were exposed to a deficient irrigation regime (W−E+), they did not show significant differences compared to those well-watered without inoculation (W+E−), and interestingly, inoculated plants showed a robust increase in shoot biomass under well-watered condition (W+E+) compared to the non-inoculated (W+E−) (Figure 2). The inoculated strawberry plants (W+E+) displayed a higher shoot biomass production, increasing by about 38% compared to those well-irrigated plants without endophytes (W+E−). The biomass production was strongly reduced when non-inoculated plants were exposed to a deficient irrigation treatment (W−E−), but the biomass production increased close to 51% in the inoculated plants under water deficiency (W−E+) (See Table 1). Additionally, plant survival also increased under drought conditions mainly due to the presence of the fungal endophytes (Table 1).

**TABLE 1:** Shoot and root fresh biomass of strawberry plants.

Parameter	W+E−	W+E+	W−E−	W−E+
Shoot fresh biomass (g)	4.98 ± 0.72 ^b^	6.92 ± 0.72 ^a^	2.12 ± 0.49 ^c^	4.09 ± 0.55 ^b^
Root fresh biomass (g)	6.99 ± 0.67 ^a^	7.55 ± 0.82 ^a^	4.03 ± 0.96 ^b^	6.29 ± 1.19 ^a^
Survival (%)	67.6	71.4	42.9	85.7

Biomass and survival of strawberry plants were evaluated after 60 days of treatment. Plants were well-watered and deficient irrigated (W+ and W−, respectively) and with or without the presence of fungal endophytes (E+ and E−, respectively) isolated from Antarctic plants. Different letters indicate significant differences (*p* < 0.05).

### Yield and fruit color of strawberry plants

Concerning fruit color and number, the effect of fungal endophytes in well-watered plants vs. plants under drought stress showed a clear addition-yield response (Table 2). The incidence of endophytes addition (E+) in the production showed a positive impact; even when the fruit number was higher in well-watered plants without endophytes (W+E−), the weight parameter between well-watered plants with or without endophytes (W+E− and W+E+) and plants exposed to deficient irrigation with endophytes (W−E+) did not show significant differences between them. Fruit production in uninoculated and drought-exposed plants (W−E−) was significantly decreased compared to the other three conditions. With respect to the quality parameters evaluated, interesting results on the diameter of fruit were evidenced in deficient irrigated plants (W−), where, the addition of endophytes increases the diameter values significantly, reinforcing the best performance obtained in plants when watering conditions are restricted. With respect to the color quality evaluated, redness (a*) and yellowness (b*) exhibited similar values, however a high variability on standard deviation was recorded in plants deficient irrigated without endophytes (W−E−). In the case of the lightness (L*), fruit from inoculated and well-watered plants showed a significant increase in this parameter evaluated compared to the other conditions.

**TABLE 2:** Productive and quality parameters of strawberry plants.

			CIELAB				
	Fruit number	Weight (g)	L	a	b	Diameter (mm)	Color
W+E−	40	5.27 ± 1.30^a^	26.45 ± 3.70^b^	32.70 ± 4.42^a^	16.00 ± 5.24^a^	13.90 ± 2.38^a^	
W+E+	30	5.54 ± 1.95^a^	29.23 ± 1.35^a^	31.85 ± 4.73^a^	15.72 ± 3.42^a^	13.31 ± 3.26^a^	
W−E+	23	4.52 ± 1.96^a^	26.40 ± 2.28^b^	32.97 ± 4.89^a^	18.62 ± 5.10^a^	11.43 ± 1.34^b^	
W−E−	7	3.44 ± 0.11^b^	27.98 ± 13.02^b^	33.12 ± 14.28^a^	18.07 ± 12.01^a^	8.67 ± 0.47^c^	

Fruit number, weight, skin fruit color, and diameter of strawberry plants were evaluated after 60 days of treatment. Plants were well-watered and deficient irrigated (W+ and W−, respectively) and with or without the presence of fungal endophytes (E+ and E−, respectively) isolated from Antarctic plants. Different letters indicate significant differences (*p* < 0.05).

### Physiological and biochemical determinations in inoculated strawberry plants under drought stress

**FIGURE 3:**
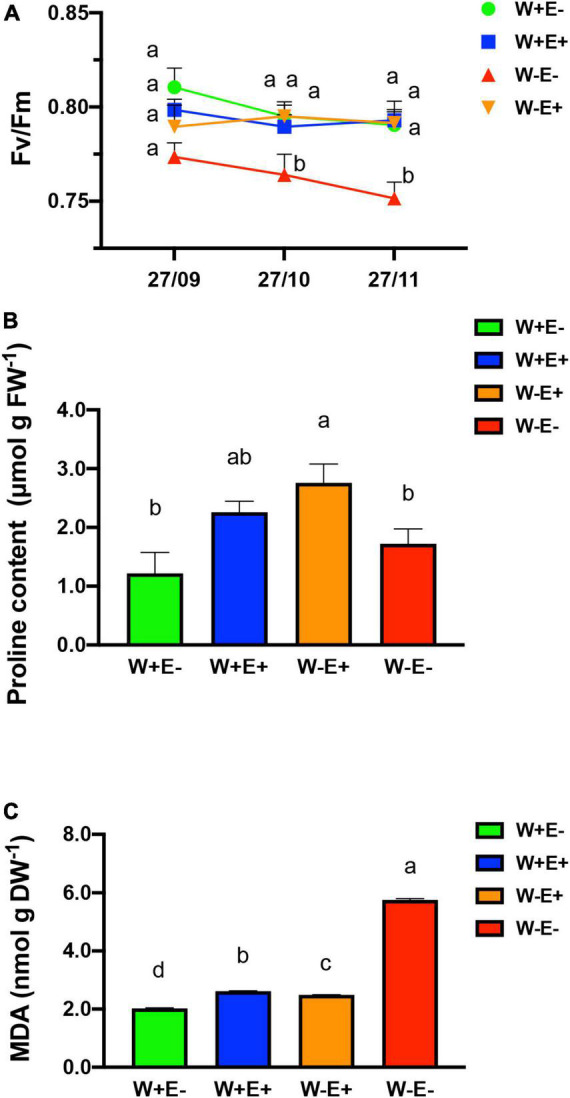
Physiological and biochemical effects over plant mediated by endophytic fungi. **(A)** Photosynthetic capacity in strawberry mediated by the presence of endophytic fungi; **(B)** proline concentration in plants, as a molecular marker of the osmoprotective response; **(C)** lipid peroxidation analysis. W–E–: without irrigation and without endophyte fungi; W–E+: without irrigation and with endophyte fungi; W+E–: with irrigation (well-watered) and without endophyte fungi; W+E+: with irrigation (well-watered) and with endophyte fungi. Different letters indicate significant differences (*P* < 0.05; two-way ANOVA). Bars represent means ± SE from three independent experiments.

To determine whether or not the plants are subjected to stress due to the water deprivation treatment, we carried out three different tests. First, to determine the integrity of the plants, the ecophysiological parameter of the photosynthetic capacity was carried out (Figure 3A). Fv/Fm, is a parameter of the potential quantum efficiency of PSII, related to plant photosynthetic performance; when photosynthetic apparatus of plants is under environmental stresses, this value will be found below to 0.80. The result showed that water deprivation treatment negatively affected the photochemical efficiency of strawberry plants without endophytes (W−E−). On the contrary, inoculated plants exposed to drought (W−E+) did not show significant differences to those plants with optimal irrigation regimes (W+E− and W+E +) after 60 days of treatment.

With regard to the plant pigments, total chlorophyll content increased in inoculated plants under water deficit (W−E+) compared to uninoculated plants under water deficit (W−E−). No significant differences were observed between inoculated well-watered (W+E−), uninoculated well-watered (W+E−), and uninoculated plants exposed to drought (W−E−) (Table 3). The same behavior was observed in chlorophyll *b* content, indicating that water availability did not affect significantly the total chlorophyll and chlorophyll *b* content. In contrast, when chlorophyll *a* was evaluated, the content was significantly increased in plants with endophytes and exposed to deficient irrigation (W−E+) compared to those without endophytes under drought stress (W−E−) and also to well-watered and uninoculated plants (W+E−). Finally, carotenoid content was evaluated, and the results obtained did not show significant differences between water treatments and plant inoculation (Table 3).

**TABLE 3:** Chlorophylls and carotenoid content of strawberry plants.

Parameter (μg/mL)	W+E−	W+E+	W−E−	W−E+
Total chlorophyll	10.84 ± 0.75^ab^	12.47 ± 3.15^ab^	9.69 ± 0.96^b^	14.56 ± 2.61^a^
Chlorophyll *a*	7.69 ± 0.54^b^	8.96 ± 2.31^ab^	6.90 ± 0.67^b^	10.49 ± 1.78^a^
Chlorophyll *b*	3.15 ± 0.20^ab^	3.52 ± 0.84^ab^	2.79 ± 0.28^b^	4.07 ± 0.83^a^
Carotenoids	2.74 ± 0.23^a^	2.94 ± 0.77^a^	2.57 ± 0.48^a^	3.25 ± 0.55^a^

Chlorophylls and carotenoid content of strawberry plants were evaluated after 60 days of treatment. Plants were well-watered and deficient irrigated (W+ and W−, respectively) and with or without the presence of fungal endophytes (E+ and E−, respectively) isolated from Antarctic plants. Different letters indicate significant differences (*p* < 0.05).

As an additional biochemical approach to evaluate the effects of water deprivation stress and Antarctic fungal endophytes, the free proline content was determined as a response molecule indicative of stress level (Liang et al., 2013). For this reason, we evaluated the proline content in different conditions with and without endophytic fungi (Figure 3B). The result of the proline level assessment showed a significant increase in plants with endophytes under drought stress (W−E+) compared with plants without endophytic fungi after 60 days of hydric deficit treatment (W−E−) (Figure 3B). In well-watered plants with endophytes (W+E+) or without endophytes (W+E−), the proline accumulation did not show significant differences compared to plants exposed to deficiently irrigated and without endophytes (W−E−).

The oxidative degradation of the lipid was also monitored to evaluate the protective role of fungal endophytes in plants exposed to drought. We evaluated the cell membrane lipid peroxidation (Figure 3C). The result showed that the presence of endophytes improved the tolerance to water deficit. Under water deficit, plants with fungal endophytes (W−E+) display a significant decrease in the MDA content compared to the plants without endophytes (W−E−). The MDA accumulation in plants under water deficit show significant differences compared to plants with normal irrigation when fungal endophytes are present, and when the endophytes are absent observed even lower decreased values were observed (Figure 3C).

### Antioxidant enzymatic activity of plants

**FIGURE 4:**
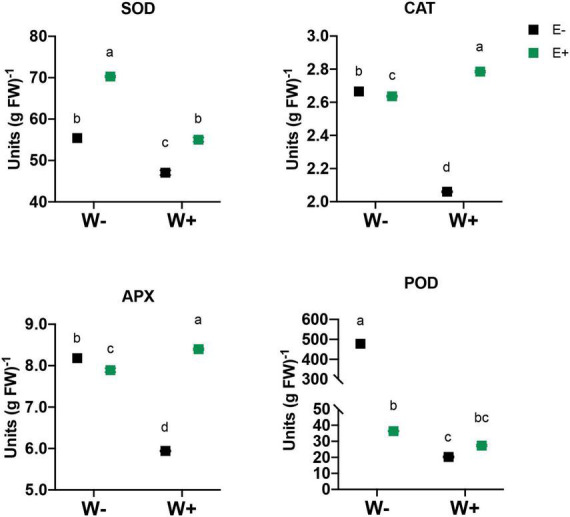
Effect of fungal Antarctic endophytes and water deficit treatments on the activities of the antioxidant enzymes (CAT, POD, SOD, and APX) in the leaves of strawberry plants. Different letters indicate significant differences (*P* < 0.05; two-way ANOVA). Bars represent means ± SE from three independent experiments.

With respect to these effects on the antioxidant enzymatic activity triggered by drought, SOD, CAT, APX, and POD activity were evaluated in leaves from inoculated and uninoculated strawberry plants (Figure 4). The enzymatic activity was lower in well-watered plants without endophyte (W+E−) compared to other evaluated treatments (W+E+, W−E+ and W−E+). In the case of inoculated plants, SOD increased its activity in well-watered (W+E+) and also in plants exposed to drought (W−E+) compared to uninoculated plants (W−E−). Meanwhile for CAT and APX, the enzymatic activity decreased in inoculated plants exposed to drought (W−E+), but the activity increased in well-watered inoculated plants (W+E+) compared to the uninoculated (W+E−). The activity of POD showed a strong decrease in inoculated plants under drought stress (W−E+) and a significant decrease in well-watered plant uninoculated (W+E−) plants but a slight increase was detected when compared with inoculated well-watered plants (W+E+) (Figure 4).

## Discussion

In this work, we tested the effects of the functional symbiosis between Antarctic fungal endophytes with strawberry plants, which is well documented stating that deficient irrigation stress shows a detrimental performance in its physiological parameters and triggers a reduction in yield (Mozafari et al., 2019; Cai et al., 2020). We noted that symbiosis is effective in the roots of well-watered and also drought stressed strawberry plants for at least 60 days (Supplementary Figure 1). The observations indicate that Antarctic fungal endophytes had the ability to colonize the well-watered and also drought-stressed strawberry root cells. In productive and quality parameters, this effective symbiosis was reflected with an increase in yield and maintenance in the fruit size when comparing inoculated plants to uninoculated plants grown under drought stress conditions (Table 2). The color was only affected in fruits obtained from inoculated and well-watered plants, in which a moderate but statistically significant increase in the lightness was observed compared to the other treatments. These results could be explained by the role of Antarctic fungal endophytes to enhance nutrient uptake, including nitrogen and phosphorus (Newsham, 2011; Acuña-Rodríguez et al., 2020). The symbiotic interactions increase the availability of essential nutrients for the synthesis of amino acids and proteins in the host plants (Harman et al., 2021). The protective role of Antarctic fungal endophytes has been largely documented in native host plants (Ramos et al., 2018; Barrera et al., 2020; Hereme et al., 2020; Morales-Quintana et al., 2021), as well as in commercial crop species such as lettuce, peppers, and tomato (Molina-Montenegro et al., 2016; Acuña-Rodríguez et al., 2019). Considering the results presented here, in addition to those previously reported, the symbiotic and positive effects on different plant host species could suggest that the two *Penicillium* strains isolated from Antarctic plants are promiscuous mutualists in their plant host selection.

The observed results suggest a positive role of this effective root inoculation on ecophysiological, biochemical, and yield performance on strawberry plants exposed to deficient irrigation regimes and in some parameters even higher than under well watered conditions. Endophyte-inoculated plants showed a higher photochemical efficiency (Fv/Fm), even when the plants were exposed to water restriction. This could be improving the efficiency of excitation energy capture by chloroplasts and the energy cycling among photosynthetic apparatus in strawberry plants under drought stress, similar to that observed in *Ceratonia siliqua* L (Boutasknit et al., 2021). The lowest value of Fv/Fm observed in plants under water stress and non-inoculated (W−E−) could be associated with an increase in energy dissipation in the PSII antennae previously reported by Demming-Adams et al. (1993) as a common response of photosynthetic apparatus to environmental stress. Additionally, drought stress leads to a decrease in chlorophyll content reducing the photosynthetic efficiency of plants (Astolfi et al., 2012). Based on this, the estimation of chlorophyll content is an effective proxy to assess the plant performance under stress. The results showed that the inoculation with Antarctic fungal endophytes improved the chlorophyll concentration of strawberry plants under drought stress (Table 3) in accordance to previous reports on the inoculation with fungal endophytes that improved plant chlorophyll concentration under drought stress (Ghorbani et al., 2018). The positive impact of inoculation in yield and photosynthetic pigments could be related with a higher osmotic pressure associated to proline accumulation, and this role has been related previously with an increase in the absorption of water and nutrients, both processes related with photosynthesis and yield in plants (Mafakheri et al., 2010). For other side, the similar values observed in carotenoids in all treatment could be attributed to the protective role and protect chlorophyll from photo-oxidation (Young, 1991).

Free proline content, on the other side, is an amino acid that normally accumulates in plants under drought and salt stress to improve their tolerance (Kishor et al., 2005). Several studies associate the plant response to drought to its role as an osmolyte, and others also indicate that the proline acts as a free-radical scavenger, membrane stabilizer, and regulating the redox potential of cells (Verbruggen and Hermans, 2008; Hayat et al., 2012). The free proline accumulation in plant cells is a phenomenon that is well known to be triggered under water deficit (Hare et al., 1998). Our results show an increase in free proline content in endophyte-inoculated plants under water stress (W−E+) when compared with the other treatment evaluated to exhibit the protective activity of endophyte when restrictive water conditions are applied; however, no significant difference between all treatment was observed; similar result was reported by Sun et al., 2015 when strawberry plants were exposed to mild drought stress and compared to control plant without water restriction. Similar results were observed by Kavroulakis et al. (2018) when tomato plants were subjected to water restriction and evaluated the effect of *Fusarium solani* FsK, another root endophyte. In addition, lipid peroxidation is considered as a hallmark of cell damage, and here it was estimated by evaluating the concentration of MDA using the assay of thiobarbituric acid reactive substances (TBARS) (Egert and Tevini, 2002). Respect to MDA content, a reliable marker for determining the degree of injury to a stressed plant (Morales and Munné-Bosch, 2019), a lower level of oxidative stress (MDA) in inoculated compared to uninoculated plants was observed. This result is an indicative parameter of the protective role exerted by the fungal endophytes on plants under drought stress (Figure 3). The results are in accordance with previous observations in the Antarctic plant *C. quitensis* under a water stress scenario that is likely to be provoked by the global climate change (Hereme et al., 2020; Morales-Quintana et al., 2021).

Reactive oxygen species are oxidizing species, where the hydroxyl radicals (OH) and singlet oxygen (O_2_) are the most important molecules. These species are very powerful oxidants that can react with all the components of living cells, producing severe damage to organic macromolecules (lipids, proteins, and nucleic acids) under oxidative stress situations (Sies, 2014). The effect in plant has been related to photosynthesis and photoinhibition, as well as to the environmental responses of photosynthesis (Del Rio, 2015). As is well documented, drought provokes the leakage of electrons during photosynthesis and respiration that generates cellular oxidative stress enhancing the production of reactive oxygen species (ROS) (Sánchez-Rodríguez et al., 2012). To deal with the cytotoxic effects of ROS generated, plants have developed several antioxidant systems, including SOD, CAT, POD, and APX (Rahnama and Ebrahimzadeh, 2005; Simova-Stoilova et al., 2008; Erdogan et al., 2016). The inoculation with fungal endophytes affected plant physiology and biochemistry (Figure 3), and these positive effects were evident in the growth performance of the inoculated plants (Table 1). Regarding the antioxidative activity of enzymes, the results are in agreement with previous reports, in which the activities of SOD, CAT, APX, and POD increased under water-deficit stress in the plants (Wu et al., 2006; Erdogan et al., 2016), and this behavior has been related with the scavenging of ROS (Singh et al., 2020). SOD is the first enzyme involved in defense against ROS, and their function is to turn superoxide radical (O_2_^–^) into O_2_ and H_2_O_2_ (Alscher et al., 2002). These free radicals can react spontaneously with organic molecules provoking membrane lipid peroxidation, protein oxidation, enzyme inhibition, and DNA and RNA damage (Vinocur and Altman, 2005).

Here, we also observed that strawberry plants inoculated with Antarctic fungal endophytes showed high SOD activity, which contributed to enhance plant protection against drought stress. In specific, only SOD enzyme increases its activity in inoculated plants under water deficit and also in plant well-watered (Figure 4). Similar results for SOD were reported in drought stress assays in strawberry plants, where SOD was the most effective enzymatic response (Sekmen Cetinel et al., 2021). In the case of CAT, APX, and POD, inoculated plants under drought stress showed a decrease in activity, suggesting that involved molecules could be less affected compared with uninoculated-stressed plants or maybe could be related such that the fungal inoculation not only stimulates their enzymatic defense system but also stimulates a minor production of the stress metabolites.

## Conclusion

Here, we observed that it is possible to improve the performance of strawberry plants under water-stress conditions through inoculation of roots with Antarctic fungal endophytes. As was observed previously in Antarctic native plants and now in inoculated strawberry plants, the results here presented strongly suggest that the observed advantage triggered by fungal symbiosis can be transferable to commercial crops to face future conditions, which are a product of the imminent global change.

## Data availability statement

The original contributions presented in this study are included in the article/Supplementary material, further inquiries can be directed to the corresponding author.

## Author contributions

PR designed the experiments. MM performed the experiments. PR and LM-Q analyzed the data. PR and MM prepared the plant material. PR, LM-Q, CR, and SP wrote the article. LM-Q, RS-M, AC-A, CR, SP, and PR reviewed the manuscript. All authors contributed to the article and approved the submitted version.

## Footnotes

1
http://www.fao.org/home/en/

2
http://www.odepa.gob.cl/

3
https://www.ncbi.nlm.nih.gov/genbank/

4
https://www.nixsensor.com/free-color-converter/
