# Supplementary material for: Improvement in the physiological and biochemical performance of strawberries under drought stress through symbiosis with Antarctic fungal endophytes
Source: Front Microbiol. 2022 Aug 25;13:939955. doi: 10.3389/fmicb.2022.939955 (PMC9453553; doi:10.3389/fmicb.2022.939955)
Supplement: Supplementary file 1 [file Data_Sheet_1.docx]

Supplementary Material

# Supplementary Figures

**Supplementary Figure 1. Microscopical determination of the inoculated and uninoculated strawberry plants.** (A, C) Pictures of uncolonized well-watered and drought stressed strawberry roots, respectively. (B, D) Spores and hyphae growing inter- and intra-cellularly in well-watered and drought stressed strawberry roots, respectively.

# Supplementary Figures


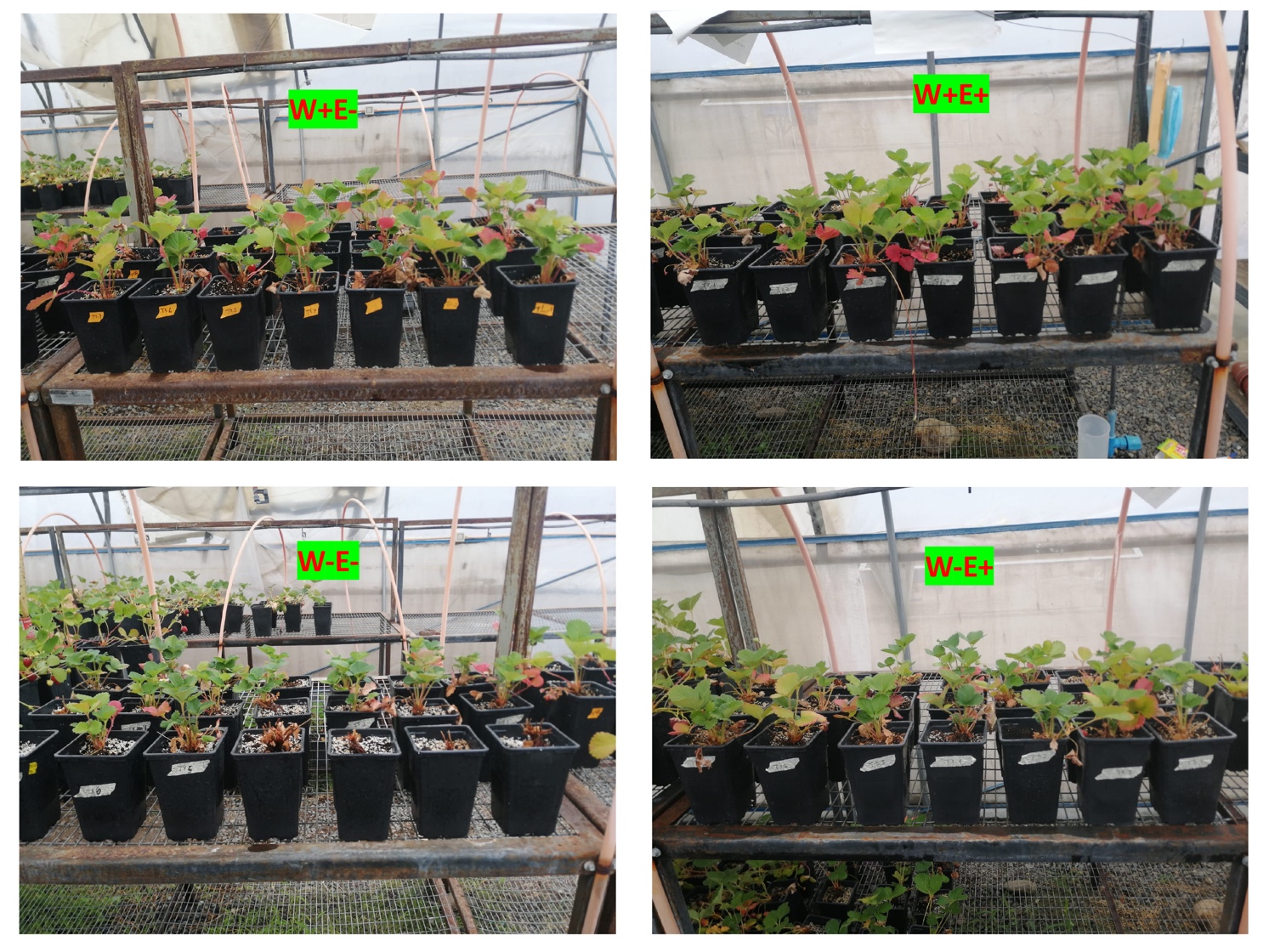


**Supplementary Figure 2.** **Strawberry plants after 60 days of drought treatment.** Uninoculated well-watered and drought stressed plants are labelled as W+E- and W-E-, respectively. Inoculated well-watered and drought stressed plants are labelled as W+E+ and W-E+, respectively.
